# BioModels’ Model of the Year 2023

**DOI:** 10.3389/fsysb.2024.1363884

**Published:** 2024-02-27

**Authors:** Rahuman S. Malik Sheriff, Hiroki Asari, Henning Hermjakob, Wolfgang Huber, Thomas Quail, Silvia D. M. Santos, Amber M. Smith, Virginie Uhlmann

**Affiliations:** ^1^ European Bioinformatics Institute, European Molecular Biology Laboratory (EMBL-EBI), Cambridge, United Kingdom; ^2^ Epigenetics and Neurobiology Unit, EMBL Rome, European Molecular Biology Laboratory, Monterotondo, Italy; ^3^ Genome Biology Unit, European Molecular Biology Laboratory, Heidelberg, Germany; ^4^ Cell Biology and Biophysics Unit, European Molecular Biology Laboratory, Heidelberg, Germany; ^5^ Quantitative Stem Cell Biology Laboratory, The Francis Crick Institute, London, United Kingdom; ^6^ Department of Pediatrics, University of Tennessee Health Science Center, Memphis, TN, United States

**Keywords:** mathematical modeling, BioModels, competition, cell cycle, protein turnover, cell-to-cell variability

## Abstract

Mathematical modeling is a pivotal tool for deciphering the complexities of biological systems and their control mechanisms, providing substantial benefits for industrial applications and answering relevant biological questions. BioModels’ Model of the Year 2023 competition was established to recognize and highlight exciting modeling-based research in the life sciences, particularly by non-independent early-career researchers. It further aims to endorse reproducibility and FAIR principles of model sharing among these researchers. We here delineate the competition’s criteria for participation and selection, introduce the award recipients, and provide an overview of their contributions. Their models provide crucial insights into cell division regulation, protein stability, and cell fate determination, illustrating the role of mathematical modeling in advancing biological research.

## 1 Introduction

The mathematical modeling of biological systems plays a crucial role in understanding complex processes, their regulation, and answering relevant biological questions. It offers a broad spectrum of industrial applications. To recognize and encourage advancements in this field, BioModels (Malik-Sheriff et al., 2020) launched the “Model of the Year 2023” competition, with supporters such as the EMBL Theory Transversal Theme, SBML, COMBINE, and ICSB. The competition aims to highlight the work of non-independent early-career researchers who have made significant contributions within the last two years (2021–2022) to the field through exciting mathematical modeling-based research. Applications were accepted from researchers including, but not limited to, PhD students, postdocs, staff scientists, and research assistants from both academia and industry. Models developed by PIs before becoming independent were also considered.

To facilitate the sharing of the model with the broader community and their potential reuse, competition participants were required to submit their models to public model-sharing repositories such as BioModels, CellCollective (Helikar et al., 2012), and Physiome (Yu et al., 2011). The Physiome repository provides a curated collection of physiological models primarily in CellML format, whereas CellCollective is a collaborative modeling building and sharing platform. BioModels is a leading repository of curated biological models which allows the submission of models in diverse modeling formats that are further manually reproduced and semantically annotated. The participants had the flexibility to submit their models in any format, including SBML, CellML, COMBINE archive, MATLAB, Mathematica, R, Python, or C++ (Malik-Sheriff et al., 2020), to enter the competition. However, participants were encouraged to submit models with well-commented or annotated code that adhered to MIRIAM guidelines (Le Novère et al., 2005).

The selection process had a strong emphasis on scientific excellence, along with the ability of the models to yield insights into complex biological phenomena or practical applications. Factors such as model reproducibility, adherence to community standards, and good code-sharing practices were also considered. The top models, regardless of their submission format—whether in a community standard like SBML or as documented code—underwent a manual verification process to ensure that their results could be reproduced. Any non-reproducible models were disqualified. Among the 25 submissions, the winning models listed below were selected on the basis of the above criteria.

• **Dr Jan Rombouts** Advisor: Gelens, L., KU Leuven, Belgium “Modular approach to modeling the cell cycle” (De Boeck et al., 2021) BioModels submission: BIOMD0000001079 BIOMD0000001080.
• **Dr Eva-Maria Geissen** Advisor: Hammarén, H.M., EMBL, Germany “Protein turnover and post-translational modification” (Hammarén et al., 2022) BioModels submission BIOMD0000001078.
• **Dr Lorenz Adlung** Advisor: Schilling M, DKFZ, Germany “Cell-to-cell variability in JAK2/STAT5 pathway” (Adlung et al., 2021) BioModels submission: BIOMD0000001077.

## 2 Overview of winning models

### 2.1 Modular approach to modeling the cell cycle

These models address the fundamental biological question of how cells regulate their division cycle. The mathematical crux of the model lies in its simulation of bistable switches, which are critical for understanding the robust and rapid transitions between different phases of the cell cycle. The models capture the essence of these bistable switches by applying a modified Hill-type ultrasensitive response. This approach allows the exploration of how cellular mechanisms, like the accumulation and degradation of cyclin B, govern the cell cycle. This approach is illustrated in two models: (1) the early embryonic cell cycle of *Xenopus laevis* (BIOMD0000001079) and (2) the somatic cell cycle with different cell cycle phases (BIOMD0000001080). The models effectively decipher the intricate balance and feedback loops involved in cell cycle regulation and have the potential to offer a profound understanding of cell proliferation and its dysregulation in diseases.

### 2.2 Protein turnover and post-translational modification

This model helps us understand whether protein turnover data from metabolic labeling experiments can reveal the impact of post-translational modifications (PTMs) on protein stability. Through its reaction rate equations framework, the model dissects the influence of the dynamics of interconvertible proteo-forms—different forms of the same protein differentiated only by their PTMs—on the measured protein turnover dynamics. The model revealed that these dynamics mask the actual stability-related dynamics of proteins. However, the model highlighted the order of PTM addition and/or removal relative to protein synthesis. This insight is vital to the accurate interpretation of PTM-resolved turnover data and an understanding of protein modification in the context of its lifecycle.

### 2.3 Cell-to-cell variability in JAK2/STAT5 pathway

This model addresses the crucial question of how erythroid progenitor cells decide between proliferation, differentiation, and apoptosis. The model employs a sophisticated series of coupled ordinary differential equations to unravel the JAK2/STAT5 signaling pathway’s role in this decision-making process. The mathematical modeling here is pivotal in identifying the specific thresholds of STAT5 activation that determine cell fate and it addresses a significant gap in our understanding of erythropoiesis. The model’s strength lies in its ability to handle the inherent cell-to-cell variability within a population, providing insights critical for developing targeted therapies for blood disorders.

## 3 Discussion

Through the “Model of the Year 2023” competition and its next edition, “Model of the Year 2024”, BioModels aims to recognize outstanding contributions from early-career researchers to systems biology modeling and highlight the crucial role that these models play in answering fundamental biological questions or industrial applications. Each winning model exemplifies how mathematical modeling can be harnessed to dissect complex biological processes, providing insights that are pivotal for both basic biological understanding and potential therapeutic applications. These models are testament to the potential of integrating mathematical modeling with biological research.

## Data Availability

The codes of models presented in the article are publicly available in the BioModels repository; further inquiries can be directed to the corresponding author.
